# Our Clinical Experience With Patients Requiring Intensive Care for Tracheal Stenosis: A Retrospective Case-Control Study

**DOI:** 10.7759/cureus.45978

**Published:** 2023-09-26

**Authors:** Umit Murat Parpucu, Semih Aydemir

**Affiliations:** 1 Anesthesiology and Reanimation, Gulhane Faculty of Medicine - University of Health Sciences, Ankara, TUR; 2 Anesthesiology and Reanimation, Ankara Atatürk Chest Diseases and Thoracic Surgery Training and Research Hospital, University of Health Sciences, Ankara, TUR

**Keywords:** intensive care, interventional bronchoscopy, post tracheostomy, post intubation, tracheal stenosis

## Abstract

Background and objective

The clinical course in patients with tracheal stenosis (TS) ranges from being asymptomatic to respiratory failure requiring follow-up in the ICU. In this study, we aimed to assess the clinical characteristics, management, and outcome of TS patients who were admitted to the ICU.

Materials and methods

The data of patients hospitalized in the ICU due to TS between January 01, 2015, and January 01, 2016, were analyzed. The patients were classified into two groups: the post-intubation tracheal stenosis (PITS) group and the post-tracheostomy tracheal stenosis (PTTS) group. Demographic characteristics, body mass index (BMI), the Acute Physiology and Chronic Health Evaluation II (APACHE II) score and the Sequential Organ Failure Assessment (SOFA) score of patients, factors that caused TS, management of TS, and ICU data of patients were compared. The outcome measures of our study were the ICU management of patients diagnosed with PITS or PTTS, their clinical characteristics, and differences in the treatment between patients diagnosed with PITS and those with PTTS in the ICU.

Results

Fifteen (75%) patients had PITS and five (25%) had PTTS. While BMI was significantly lower in patients in the PTTS group, the APACHE II and SOFA scores were significantly higher in PTTS patients (p<0.05). In most of the patients in the PITS group, the location of the stenosis was subglottic and at the 1/3 upper part of the trachea, while in the PTTS group, it was located only at the upper 1/3 of the trachea (p>0.05). Mechanical dilatation was performed in all patients in both groups. Mechanical dilatation and cryotherapy were performed in 10 (66.7%) patients in the PITS group (p>0.05), and a stent was applied in addition to this treatment in three (20%) patients in the PITS group and four (80%) patients in the PTTS group (p<0.05). Mechanical ventilation was not needed in 10 (66.7%) PITS patients and three (60.0%) PTTS patients after the interventional procedure. All patients were eventually discharged from the ICU after treatment.

Conclusion

While higher BMI was common in PITS patients, the PTTS patients were generally in worse condition. In this patient group, interventional pulmonology procedures in the ICU can be life-saving.

## Introduction

Tracheal stenosis (TS) is an uncommon but life-threatening condition. Despite major advances in medicine, it is still a significant concern in clinical practice. The etiology of TS is frequently associated with conditions such as congenital diseases, post-intubation tracheal stenosis (PITS), post-tracheostomy stenosis (PTTS), tracheal tumor, and external compression on the trachea [1,2]. Despite the scarcity of data regarding TS incidence, acquired TS is often linked to PITS, with a reported incidence ranging from 0.6% to 21% [3]. The estimated annual incidence of PITS and PTTS cases in the general population is 4.9 cases per million [1]. While TS develops due to compression caused by the intubation tube cuff in PITS patients, it is caused by tracheotomy stoma and tracheal cartilage in patients with PTTS [4].

Depending on the severity of the stenosis in TS, patients may manifest conditions ranging from being asymptomatic to respiratory failure [3]. The clinical conditions of TS patients may deteriorate rapidly and they may often need ICU management. Due to TS and the comorbidities that accompany it, the management of these patients in the ICU can be quite challenging for intensivists. Besides ICU follow-ups, many interventional pulmonology procedures are also performed in these patients [5]. Interventional bronchoscopy procedures are a preferred method used before surgical treatment due to their curative benefits for many patients, including those who are candidates for surgery [6]. Even though most of the patients benefit from these interventions, some patients may require re-admission to the ICU and repeat interventional procedures. A comprehensive clinical evaluation and a multidisciplinary approach often lead to positive outcomes in these patients.

In this retrospective case-control study, we aimed to highlight the clinical features, management, and ICU treatment outcomes among 20 consecutive patients admitted to the ICU with a diagnosis of either PITS or PTTS.

## Materials and methods

Study population and design

This retrospective case-control study was conducted after obtaining approval from the Ethical Committee (protocol number: 2012-KAEK-15/2503; dated 12/04/2022). The Committee did not require informed consent as the study was retrospective in design. All procedures followed were in accordance with the ethical standards (institutional and national) of the committee responsible for human experiments and the Declaration of Helsinki, as revised in 2013.

The data of patients over the age of 18 years who were diagnosed with PITS and PTTS in the ICU between January 01, 2015, and January 01, 2016, were included in the study. Data of patients with malignancy and idiopathic causes of TS were excluded from the study. The patients were attended to by the same anesthesiologist in the anesthesiology and reanimation ICU of Ankara Atatürk Sanatorium Training and Research Hospital (formerly known as Atatürk Chest Diseases Thoracic Surgery Training and Research Hospital). The patients were classified into two groups: the PITS group and the PTTS group.

Patients' age, gender, body mass index (BMI), comorbidities, the Acute Physiology and Chronic Health Evaluation II (APACHE II) and Sequential Organ Failure Assessment (SOFA) scores, causes of TS, length of stay (LOS) in the ICU, TS-related symptoms, the degree of TS, localization of TS, treatment modality for TS, complications after TS treatment, and ICU mortality were recorded. BMI as well as APACHE II and SOFA scores were recorded from the patients' ICU admission data. Also, the duration of intubation was recorded for patients in the PITS group, and the duration of tracheostomy was recorded for patients in the PTTS group. The data were compared between the groups.

Clinical definitions

TS was considered in patients with a history of long-term intubation or tracheostomy who were hospitalized in our ICU with respiratory failure. The diagnosis of TS was made by thoracic and cervical CT or bronchoscopy. The classification defined by Myer et al. [7] was used to compare the diameter of the tracheal lumen with the bronchoscope: lumen up to 50% was classified as grade I, 51%-70% as grade II, and over 70% as grade III. An airway with no visible lumen was defined as grade IV. Laryngotracheal stenosis localization was classified into localization in the glottis, subglottic area, 1/3 of the upper tracheal area, 2/3 of the upper tracheal area, and two localizations or more.

Outcome measures

The primary outcome of our study was the ICU management of patients diagnosed with PITS or PTTS. The secondary outcomes were the clinical characteristics and differences in the treatment between patients diagnosed with PITS and those with PTTS in the ICU.

Statistical analysis

The IBM SPSS Statistics version 23.0 (IBM Corp., Armonk, NY) was used for statistical analysis of the gathered data. Categorical variables were presented as numbers and percentages. Continuous variables were presented as mean, standard deviation (SD), and ranges. The compatibility of the variables to normal distribution was examined using the Kolmogorov-Smirnov/Shapiro-Wilk tests. The Chi-square test and Fischer's exact test were used for comparisons of categorical variables. The independent student's t-test was used in groups with normal distribution and the Mann-Whitney U Test was used in groups that did not comply with normal distribution. A p-value <0.05 was considered statistically significant.

## Results

**Figure 1:**
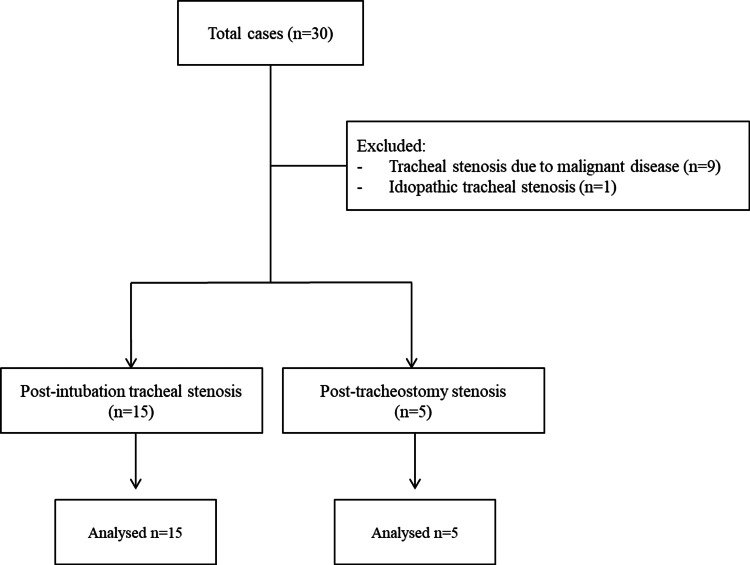
Flow chart depicting the selection of patients

Between January 01, 2015, and January 01, 2016, a total of 30 adult patients aged 18 years and over were hospitalized with a diagnosis of TS in the ICU. The data of patients with malignancy and idiopathic causes were excluded. Ultimately, the data of 20 patients were deemed eligible for analysis. Nine patients had TS related to malignant disease and one patient had idiopathic TS (Figure 1).

Fifteen (75%) patients had PITS and five (25%) had PTTS. There was no significant difference between the groups in terms of age, gender, comorbid diseases, and mean LOS in ICU (p>0.05). While BMI was significantly lower for patients in the PTTS group, APACHE II and SOFA scores were significantly higher among PTTS patients (p<0.05) (Table 1).

**Table 1:** Demographic and clinical characteristics of patients *P<0.05 PITS: post-intubation tracheal stenosis; PTTS: post-tracheostomy tracheal stenosis; BMI: body mass index; APACHE II: Acute Physiology and Chronic Health Evaluation II; SOFA: Sequential Organ Failure Assessment; LOS: length of stay; ICU: Intensive care unit; CAD: coronary artery disease; COPD: chronic obstructive pulmonary disease; DM: diabetes mellitus; SD: standard deviation

Variables	PITS (n=15)	PTTS (n=5)	P-value
Age, years, mean ± SD	56.48 ± 16.05	52.65 ± 14.67	0.595
Gender, M/F, n (%)	10 (66.7)/5 (33.3)	3 (60)/2 (40)	1.000
BMI, kg/m^2^, mean ± SD	28.33 ± 5.96	19.83 ± 3.18	0.002*
APACHE II score, mean ± SD	16.76 ± 2.55	24.83 ± 3.12	<0.001*
SOFA score, mean ± SD	4.39 ± 1.82	5.97 ± 1.81	<0.001*
LOS in ICU, days, mean (min-max)	3.54 (2-30)	5.16 (2-17)	0.503
CAD, n (%)	5 (33.3)	1 (20)	1.000
COPD, n (%)	9 (60)	2 (40)	0.617
DM, (n / %)	5 (33.3)	0 (0.0)	0.266
Hypertension, n (%)	6 (40)	0 (0.0)	0.260
Multiple comorbidities, n (%)	9 (60)	1 (20)	0.303

The cause of TS in the vast majority of patients in both groups was found to be acute respiratory failure that had developed on the basis of chronic respiratory failure and required intubation. The mean intubation time was 14.38 (4-33) days in the PITS group, and the duration of the tracheostomy was 73.33 (30-180) days in the PTTS group. For most patients in the PITS group, the location of the stenosis was subglottic and at the 1/3 upper part of the trachea, while in the PTTS group, it was located only at the upper 1/3 of the trachea (p>0.05). When the patients were evaluated with regard to the stenosis rate, all of them had stenosis over 50%, and the stenosis level was found to be 71%-99% in the majority of patients in both groups (Table 2).

**Table 2:** Characteristics of tracheal stenosis in terms of days of intubation, the location of the stenosis, and the degree of the stenosis *Location of tracheal stenosis: 1: lesion with glottis involvement, 2: 1/3 of the upper tracheal part, 3: midtracheal location, 4: 1/3 of the lower tracheal part. **Degree of stenosis is defined according to the Myer-Cotton subglottic stenosis grading scale [7] - grade 1: 0-50%, grade 2: 51-70%, grade 3: 71-99%, and grade 4: complete stenosis PITS: post-intubation tracheal stenosis; PTTS: post-tracheostomy tracheal stenosis

Variables	PITS (n=15)				PTTS (n=5)			
Duration of intubation, days, mean (min-max)	14.38 (4-33)				-			
Duration of tracheostomy, days, mean (min-max)	-				73.33 (30-180)			
Location of tracheal stenosis*, n (%)	1	2	3	4	1	2	3	4
	1 (6.7)	6 (40)	6 (40)	2 (13.3)	-	1 (20)	4 (80)	-
Degree of tracheal stenosis**, n (%)	1	2	3	4	1	2	3	4
	-	3 (20)	11 (73.3)	1 (6.7)	-	1 (20)	3 (60)	1 (20)

While mechanical dilatation was performed in all PITS and PTTS patients, mechanical dilatation and cryotherapy were performed in 10 (66.7%) patients in the PITS group and one (20%) in the PTTS group. Stent was applied in addition to these procedures in three (20%) PITS patients and four (80%) PTTS patients (Table 3).

**Table 3:** Distribution of interventional treatments applied to patients *Comparison of the two groups in terms of cryotherapy treatment. **Comparison of the two groups in terms of treatment with stent PITS: post-intubation tracheal stenosis; PTTS: post-tracheostomy tracheal stenosis

Treatment	PITS, n (%) (n=15)	PTTS, n (%) (n=5)	P-value
Mechanical dilatation	2 (13.3)	-	
Mechanical dilatation + cryotherapy	10 (66.7)	1 (20)	1.000*
Mechanical dilatation + cryotherapy + stent insertion	3 (20)	4 (80)	0.031**

Mechanical ventilation was not required after the interventional procedure in 10 (66.7%) PITS patients and three (60.0%) PTTS patients. All of our patients were eventually discharged from the ICU (Table 4).

**Table 4:** ICU follow-up results of the patients PITS: post-intubation tracheal stenosis; PTTS: post-tracheostomy tracheal stenosis; ICU: intensive care unit; MV: mechanical ventilation

Results	PITS (n=15)	PTTS (n=5)	P-value
Patients who did not need MV after the procedure, n (%)	10 (66.7)	3 (60.0)	1.000
Discharged from the ICU, n (%)	15 (100)	5 (100)	

## Discussion

Based on our findings, PTTS patients had higher APACHE II and SOFA scores and lower BMI values. Interventional procedures were also different in both groups. Although the patient data is limited, particularly in the PTTS group, our study differs from similar studies as we included critical patients who needed ICU care. Zias et al. [8] evaluated PITS and PTTS patients in a study where the majority of patients were obese females with a history of diabetes mellitus, hypertension, and cardiovascular diseases. In the present study, when the history of comorbidities among PITS and PTTS patients was evaluated, there was a predominance of patients with multiple comorbidities and chronic obstructive pulmonary disease (COPD). This could be attributed to the fact that our institution is a reference hospital for patients with chronic respiratory diseases, particularly COPD.

The association between gender and TS development is a controversial issue, and it has been emphasized that this situation may be associated with idiopathic TS [9-11]. In our study, we did not observe any significant differences in terms of gender. In addition, the high number of male patients in the PITS group may be related to the high number of COPD patients. BMI was higher in the PITS group and APACHE II and SOFA scores were found to be higher in the PTTS group during ICU admission. BMI was not at a morbidly obese level, and this is consistent with the findings in the literature [8]. In addition, we think that lower BMI accompanied by higher APACHE II and SOFA scores in the PTTS group may be associated with poor nutritional status and prolonged chronic processes in the PTTS patients.

There are varying results in the literature regarding the duration of stenosis development after intubation or tracheostomy [8,12]. Although there is much data about the PITS etiology in the literature, it remains a matter of controversy. Compression and ischemia-induced necrosis are thought to be significant factors as they can cause mechanical damage to the tracheal mucosa. Local infections, low blood pressure, steroid administration, sensitivity to intubation materials, and, in addition to all these factors, many individual problems may also cause TS [12-14]. All these factors may be related to the time of development of PITS. In our study, this period was found to be 14.38 days. Stenosis due to tracheostomy usually develops in the tracheostomy stoma. Cartilage damage, abnormal cartilage tissue healing, granulation tissue development, and local infections can cause stenosis. Also, prolonged intubation time before tracheotomy can play a role in the development of stenosis [10]. Usually, TS development takes more time in tracheotomies than in PITS cases. In our study, the average time for TS development was 73.33 days with the shortest being 30 days and the longest 180 days.

It has been stated in the literature that the degree of TS is generally over 50%. In addition, stenosis localization is mostly subglottic and located at the 1/3 upper level of the trachea [7,9,13]. In the present study, although the localization was similar to what is described in the literature, the level of stenosis was observed to be over 71-99% in many patients according to the Myer-Cotton subglottic stenosis grading scale [7]. This may explain why patients need urgent ICU admissions. All of our patients needed mechanical ventilation due to severe respiratory distress.

Various interventional treatment strategies have been employed for the management of patients with PITS or PTTS [4,6,8,15,16]. The location of the stenosis, the reason for TS, comorbidities, and the degree of stenosis are the main factors considered in choosing the appropriate treatment procedure. A careful assessment and multidisciplinary approach are the keys to success [4]. These become even more important in critical patients who need ICU admission. In our cases, we determined the treatment plans for the patients after their critical conditions were managed in the ICU. In both groups, before the procedure-related decisions were made, stenosis characteristics were evaluated by fiber optic bronchoscope or CT. In this study, mechanical dilatation and cryotherapy were the preferred methods in all patients, and stents were also used in three patients in the PITS group and four patients in the PTTS group. Surgery may not be suitable for a small subset of patients who develop PITS. In these patients, stents can be applied to prevent the development of life-threatening restenosis and to enhance patient comfort. This may give the patient a chance until surgery is deemed safe. Although stent placement is considered a last resort, it may be required in the presence of tracheomalacia, especially in stenoses due to tracheotomy [4,12,14]. In our study, all patients were eventually discharged from the ICU. We believe that interventional bronchoscopic procedures reduce respiratory distress and are beneficial during the discharge process.

This study has a few limitations. It was retrospective in design and conducted at a single center. In addition, the number of patients was quite different between the groups; however, this is due to the low possibility of encountering these patients. At the same time, it should be noted that some PTTS patients may also have PITS, and we did not classify the patients in this way. Additionally, there was no data regarding the tracheostomy method for all patients who developed tracheal stenosis due to tracheostomy. Moreover, there was no follow-up of our patients after discharge from the ICU. Since the number of patients in our study was small and it was a single-center study, the results cannot be generalized to the broader population; there is also a potential for selection bias, and prospective studies with a larger number of patients are required to gain more insights into the topic.

## Conclusions

The management of patients with PITS or PTTS can be very challenging in the ICU. Higher BMI is fairly common, particularly in PITS patients. Interventional pulmonology procedures are of vital importance for ICU discharge in these patients. The main benefits of interventional pulmonology procedures are that they reduce hospital stays and help avoid the necessity for ICU care. Additionally, these are less intrusive. We recommend more prospective research involving ICU patients on this topic.
